# Dynamic response of axle box bearing for high-speed train considering wheelset flexibility and polygonal wear

**DOI:** 10.1038/s41598-023-50177-2

**Published:** 2023-12-19

**Authors:** Tiantian Guan, Xiaoyu Deng, Jiangwen Wang

**Affiliations:** 1 Chengdu Aeronautic Polytechnic, Chengdu, 610100 China; 2 Urban Vocational College of Sichuan, Chengdu, 610110 China; 3 State Key Laboratory of Rail Transit Vehicle System, Southwest Jiaotong University, Chengdu, 610031 China

**Keywords:** Mechanical engineering, Nonlinear phenomena

## Abstract

In this study, a flexible wheelset was added to a rigid-flexible coupled vehicle dynamics model, in which the axle box bearings are accurately modeled. The measured wheel’s polygon wear profile and Wuhan-Guangzhou track spectrum are used in the model to define the wheel tread and track irregularity, respectively. We conducted a field test on the Wuhan-Guangzhou railway line to validate the model. Then, we investigate how the dynamic properties of the axle box bearing are impacted by the wheelset flexibility and polygonal wear of wheel. We found that the polygonal wheel with a rigid wheelset causes high-frequency vibration in wheelset and axle box, and increases the axle box bearing’s internal contact force. Additionally, the flexible wheelset with a normal wheel tread can alleviate the wheel/rail impact and reduce the axle box’s vertical vibration as well as the axle box bearing’s internal contact force. When the vehicle is running at *v* = 300 km/h, the excitation frequency caused by the wheel's 20th-order polygon is 576.5 Hz, and the flexible wheelset's 20th-order modal frequency is 577 Hz. The two frequencies are similar, when considering the polygonal wheel and flexible wheelset simultaneously, the wheelset will resonate. And the resonate of wheelset will increase the local deformation of the axle end and deteriorate the bearing operating environment, causing a significant increase in the bearing contact force. Finally, the axle box bearing’s dynamic characteristics are summarized when vehicle velocity varies from 50 to 350 km/h and wheel polygon wear amplitude ranges from 0.01 to 0.05 mm.

## Introduction

**Figure 1:**
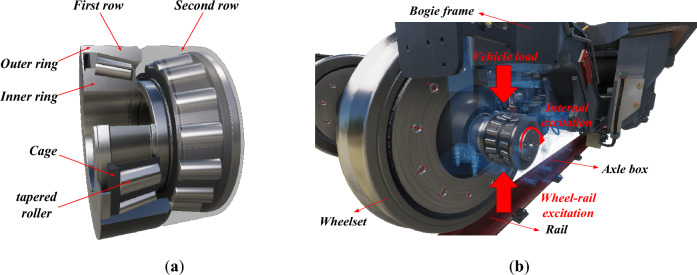
The double-row TRBs for high-speed train: (**a**) the internal structure; (**b**) the operating environment.

The axle box bearings used for high-speed trains are typically double-row tapered roller bearings (TRBs). The internal structure of TRB is shown in Fig. 1a, tapered rollers are positioned in the gap between the inner and outer rings, and the rollers are secured by the cage to maintain relative spacing. The inner ring is fastened to the axle end by interference fit, while the outer ring and axle box is fitted closely under the weight of the vehicle. Between the wheelset and the axle box, the double-row TRB is essential in transmitting relative motion and force. The operating speed of high-speed trains has significantly increased due to China’s rapid development and popularization of high-speed railways. Axle box bearings operate in a harsher environment since they are directly subjected to wheel/rail excitation, vehicle load, and the internal excitation caused by the high-speed rotation of the bearing element simultaneously, see. Fig. 1b. The dynamic responses of the double-row TRBs are extremely complex, which are the joint action of bearing nonlinear multi-body system and rail-vehicle system^1^. The train's operational safety will be influenced by the double-row TRBs' dynamic performance. Therefore, studying the double-row TRB’s dynamic characteristics in the vehicle operating environment is necessary and practical.

Vehicle system dynamics theory includes vertical dynamics^2^, lateral dynamics^3^, longitudinal dynamics^4^, and rigid-flexible coupling dynamics^5^. Early research on the vehicle dynamic system mainly focused on structural components such the car body^6,7^, bogie frame^8,9^, and wheelset^10,11^. Recently, scholars have gradually shifted their research focus to vital components including traction motors^12–14^, gear transmission systems^15,16^, and axle box bearings. For the axle box bearing, owing to the complex nonlinear geometric characteristics and nonlinear contact of the internal structure, the lumped mass model^17^, quasistatic model^18^, dynamic model^19,20^, and finite element model^21^ are usually used. To analyze roller-race interaction in detail, Jones^18^ used the quasistatic model to examine the effects of arbitrary load on ball bearings and radial roller bearings. Gupta^19,20^ presented an analytical formulation for the motion of the roller in a cylindrical roller bearing. Singh^21^ analyzed the interaction of bearing components and considered the influence of bearing defects using the finite element method. Stribeck^22^ established a static dynamic model of ball bearing under various loads. Harris^23^ extended the quasistatic model to different types of bearings and studied the contact force between cylindrical rollers and raceways when various load excitations are applied. Petersen^24^ analyzed bearings’ stiffness, contact force, and vibration characteristics with raceway defects.

In summary, the research on bearing dynamics can help us establish a theoretical foundation for our research on axle box bearings for high-speed trains. Most studies consider bearings as a separate component; however, the vibration of axle box bearings is greatly affected by the vibration of wheelsets and axle boxes. Wang^1^ studied the dynamic responses of the double-row TRB when the excitation of the traction drive system’s gear meshing and track irregularity were applied simultaneously. Liu^25^ established a dynamics model of a double-row TRB with roundness and waviness errors and analyzed how error amplitude and order affected bearing vibration. Lu^26^ proposed a detailed method for double-row TRB modeling with multitype defects. Liao^27^ proposed the slice method describe the contact state between inner ring rib and roller of double-row TRB. However, the abovementioned studies on the double-row TRB of high-speed trains did not consider the influence of elastic deformation of wheelsets^28^. Notably, under the wheels’ polygonal wear condition, the wheelset’s high-frequency vibration is intensified, and its elastic deformation cannot be ignored. There is, however, limited study on the double-row TRBs' dynamic reactions while considering wheel flexibility and tread polygon wear. Therefore, this study investigated the dynamic responses of double-row TRBs under the excitation of track irregularity, wheelset flexibility, and polygon wear of the wheel simultaneously through simulation calculation.

## Formulations of the dynamic model

### Vehicle dynamic model

**Figure 2:**
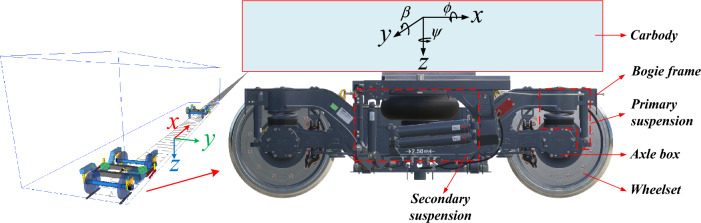
The vehicle model.

We established a vehicle dynamic model in SIMPACK, as shown in Fig. 2, which contains 15 rigid bodies, including the car body, bogie frame, wheelset, axle box, and so on. Among them, the axle box rotating arm connects the wheelset and bogie frame. Coil springs and vertical shock absorbers make up the primary suspension, whereas the secondary suspension includes air springs, lateral bump stop, traction rods, lateral shock absorbers, and antiroll bar. Additionally, the nonlinear factors of the abovementioned components were considered. According to the railway coordinate system, the vehicle’s longitudinal direction is defined as* X*-axis, and the roll angle is defined as *ϕ*. The vehicle’s lateral direction is indicated by the* Y*-axis, and its pitch angle is *β*. The vehicle’s vertical direction is indicated by the *Z*-axis, and its yaw angle is *ψ*.

**Figure 3:**
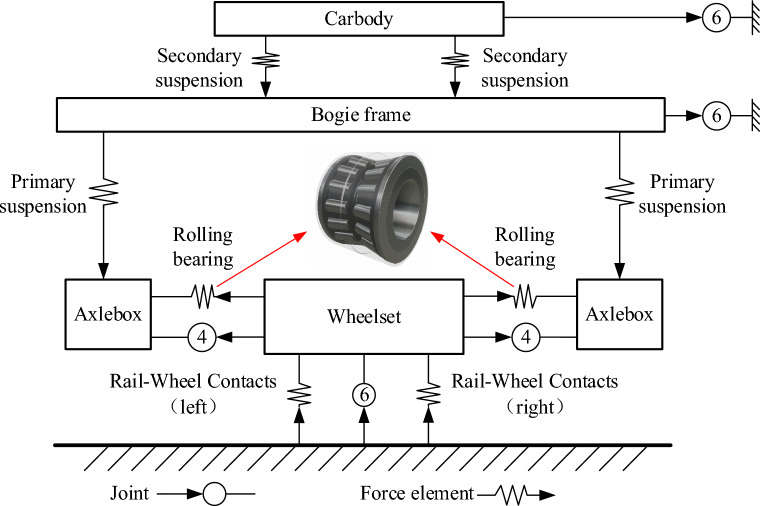
Topological model of the vehicle.

As shown in Table 1, the vehicle model contains 15 rigid bodies with a total of 74 degrees of freedom (DOFs). The car body has 6 DOFs, as well as the bogie frame and the wheelset. The wheelset’s DOFs for rolling and bouncing are constrained by wheel/rail contact. Each axle box has 4 DOFs, *Xa*, *Ya*, and *Za* present the displacements in a longitudinal, lateral, and vertical direction, and *β*_*ab*_ denotes the rotation freedom about the axle shaft. Double-row TRB forces simulate the contact forces between the axle box and shaft. A detailed description of the double-row TRB’s dynamic model is given in Section "Axle box bearing forces". Figure 3 shows the vehicle’s topological model. Table 2 shows the vehicle’s primary parameters.

**Table 1:** Lists of the vehicle model's DOFs.

Vehicle component	Type of motion					
	Longitudinal	Lateral	Vertical	Roll	Yaw	Pitch
Car body	*X*_*c*_	*Y*_*c*_	*Z*_*c*_	*ϕ*_*cb*_	*ψ*_*cb*_	*β*_*cb*_
Bogie frame (*i* = 1,2)	*X*_*bi*_	*Y*_*bi*_	*Z*_*bi*_	*ϕ*_*bfi*_	*ψ*_*bfi*_	*β*_*bfi*_
Wheelset (*i* = 1,2,3,4)	*X*_*wi*_	*Y*_*wi*_	*Z*_*wi*_	*ϕ*_*wsi*_	*ψ*_*wsi*_	*β*_*wsi*_
Axle box (*i* = 1–8)	*X*_*ai*_	*Y*_*ai*_	*Z*_*ai*_	*-*	*-*	*β*_*abi*_

**Table 2:** Primary parameters.

Items	Value
Car body mass (t)	33.8
Car body mass moment of inertia *x*/*y*/*z* (t·m^2^)	110/1656/1562
Bogie frame mass (kg)	2056
Bogie frame mass moment of inertia *x*/*y*/*z* (kg·m^2^)	1390/2590/3800
Wheelset mass (kg)	1578
Wheelset mass mass moment of inertia *x*/*y*/*z* (kg·m^2^)	840/136/840
Axle box mass (kg)	66.5
Axle box mass moment of inertia *y-axis* (kg·m^2^)	2.85
Primary suspension translational stiffness along x/y/z (kN/m)	980/980/1176
Damping of primary suspension along *z* (kN·s/m)	19.6
Secondary suspension translational stiffness along *x*/*y*/*z* (kN/m)	133/133/203
Damping of secondary suspension along *z* (kN·s/m)	10.4

### Axle box bearing forces

Table 3 shows a certain type of double-row TRB’s structural parameters. The bearing is simplified to a dynamic model only considering the contact of rollers, inner and outer rings based on the following assumptions^1^: ignoring friction and lubrication inside the bearing; pure rolling and no sliding between the rollers and raceways; the compressions along the contact line are constant, represented by *δ*_*a*_.

**Table 3:** Structural parameters of TRB.

Name	Parameter
Roller average diameter (*D*_*b*_)	23 mm
Inner ring contact angle (*α*_*i*_)	7.75 mm
Outer ring contact angle (*α*_*O*_)	10 mm
Roller effective length (*l*_*w*_)	45 mm
Roller set pitch diameter (*d*_*m*_)	180.5 mm
number of single row rollers (*z*)	21

**Figure 4:**
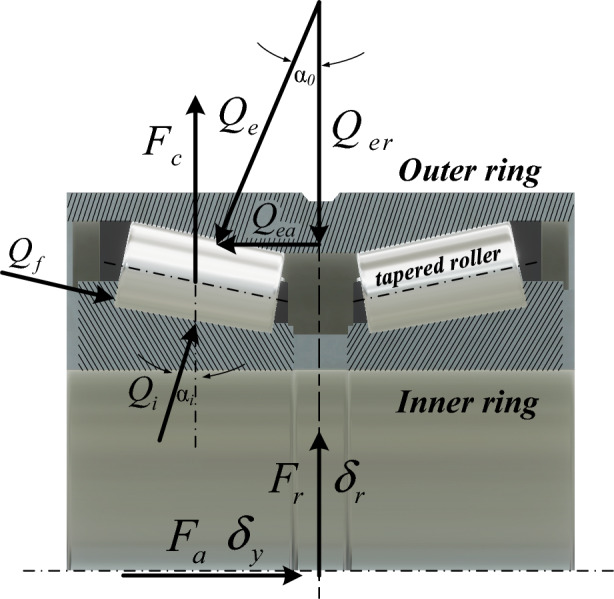
Loading of the double-row TRB.

Figure 4 shows the double-row TRB under the joint effect of a axial load *F*_*a*_ and a radial load *F*_*r*_.

Referring to Andréason^29^ and Lundberg and Palmgren^30^, the resultant contact force between the roller and the raceway along the contact line can be expressed as Eq. (1), where the value of the exponent *n* depends on the type of bearing. Based on the work of Harris and Kotzalas^31^ and similar research experience^1,25,26^, as for the TRBs used for high-speed trains, *n* = 10/9.

1 $$Q_{a} = k_{a} \delta_{a}^{n} l_{w} = K\delta_{a}^{n}$$

Assuming that the two-row bearings move in unison, the overall compression at any roller azimuth *ψ*_*i*_ may be represented as Eqs. (2) and (3) by taking into account the initial gaps *g*_*h*_ in the radial direction.

2 $$\delta_{1ei} = \cos \alpha_{0} [\sqrt {\delta_{x}^{2} + \delta_{z}^{2} } \cos \psi_{i} - 0.5g_{h} (1 - \cos \psi_{i} )] + \delta_{y} \sin \alpha_{0} (i = 1 - 21)$$

3 $$\delta_{2ei} = \cos \alpha_{0} [\sqrt {\delta_{x}^{2} + \delta_{z}^{2} } \cos \psi_{i} - 0.5g_{h} (1 - \cos \psi_{i} )] - \delta_{y} \sin \alpha_{0} (i = 1 - 21)$$

where *δ*_1ei_ and *δ*_2ei_ are the total compressions of the two-row bearings’ outer raceway contact line in the normal direction; The longitudinal, lateral, and vertical relative displacements between the inner and outer rings of the double-row TRB are *δ*_*x*_, *δ*_*y*_ and *δ*_*z*_. The contact angle between the roller and the inner and outer rings are indicated by *α*_*i*_ and *α*_*0*_, respectively.

Each roller’s contact force of the double-row TRB can be calculated by putting Eqs. (2) and (3) into (1) as follows:

4 $$Q_{nei} = \left\{ {\begin{array}{*{20}l} {K\delta_{nei}^{10/9} } \hfill & {\delta_{nei} > 0} \hfill \\ 0 \hfill & {\delta_{nei} < 0} \hfill \\ \end{array} } \right.\,\,(n = 1,2{\kern 1pt} {\kern 1pt} {\kern 1pt} {\kern 1pt} {\kern 1pt} ;{\kern 1pt} {\kern 1pt} {\kern 1pt} {\kern 1pt} {\kern 1pt} {\kern 1pt} i = 1 - 21)$$

*n* = 1, 2 respectively represent the first row and the second row of the double-row TRB, and the resultant force of the double-row TRB can be expressed as:

5 $$\left\{ \begin{gathered} F_{xa} = C_{a} (X_{a} - X_{w} - \psi_{w} a_{0} ) + \sum\limits_{n = 1}^{2} {\sum\limits_{i = 1}^{21} {Q_{nei} } } \cos a_{0} \cos \left( {\arctan \left( {\frac{{\delta_{z} }}{{\delta_{x} }}} \right)} \right)\cos \psi_{ni} \hfill \\ F_{ya} = \sum\limits_{n = 1}^{2} {\sum\limits_{i = 1}^{21} {Q_{nei} } } \sin a_{0} + C_{a} (Y_{a} - Y_{w} ) \hfill \\ F_{za} = C_{a} (Z_{a} - Z_{w} - \phi_{w} a_{0} ) + \sum\limits_{n = 1}^{2} {\sum\limits_{i = 1}^{21} {Q_{nei} } } \cos a_{0} \sin \left( {\arctan \left( {\frac{{\delta_{z} }}{{\delta_{x} }}} \right)} \right)\cos \psi_{ni} \hfill \\ \end{gathered} \right.$$

Roller azimuth location *ψ*_*i*_ can be calculated as:

6 $$\psi_{i} = \frac{2\pi }{{N_{i} }}(j - 1) + \frac{{\omega_{{{\text{ir}}}} }}{{2d_{n} }}\left( {d_{n} - D_{b} \cos \frac{{\alpha_{i} + \alpha_{e} }}{2}} \right)t$$

where *ω*_*ir*_ is the rolling angular velocity of inner ring.

The inner ring of the TRB is installed at the axle end of the wheelset, while the outer ring is fastened to the axle box. The relative displacement between the inner and outer rings of the TRB can be calculated with the following formula after considering the coupling effect of the vibration of the wheelset and axle box:

7 $$\left\{ \begin{gathered} \delta_{Lxi}^{{}} = X_{aLi} - X_{wi} - d_{w} \psi_{wi} \hfill \\ \delta_{Rxi}^{{}} = X_{aRi} - X_{wi} + d_{w} \psi_{wi} \hfill \\ \end{gathered} \right.\,\,\,\,\,(i = 1\sim 4)$$

8 $$\delta_{y(L,R)i} = Y_{wi} - Y_{a(L,R)i} \,\, (i = 1\sim 4)$$

9 $$\left\{ \begin{gathered} \delta_{Lzi}^{{}} = Z_{aLi} - Z_{wi} + d_{w} \phi_{wi} \hfill \\ \delta_{Rzi}^{{}} = Z_{aRi} - Z_{wi} - d_{w} \phi_{wi} \hfill \\ \end{gathered} \right.\mathop {}\nolimits_{{}}^{{}} \mathop {}\nolimits_{{}}^{{}} \mathop {}\nolimits_{{}}^{{}} \mathop {}\nolimits_{{}}^{{}} (i = 1\sim 4)$$

where *X*_*aLi*_, *Y*_*aLi*_, and *Z*_*aLi*_ denote the axle box’s displacements at the wheelset’s left side in the directions of longitudinal, lateral, and vertical, respectively; *X*_*aRi*_, *Y*_*aRi*_, and *Z*_*aRi*_ denote the axle box’s displacements at the wheelset’s right side in the directions of longitudinal, lateral, and vertical, respectively; *X*_*wi*_ and *Z*_*wi*_ denote the wheelset’s displacements in the directions of longitudinal and vertical, respectively;

Therefore, the double-row TRB’s contact forces can be calculated by substituting Eqs. (7)–(9) into (5).

### Wheel/rail interaction

The nonlinear Hertzian elastic contact theory determined the normal force *P*(*t*) as^32^:

10 $$p(t) = {\kern 1pt} {\kern 1pt} {\kern 1pt} [\frac{1}{G}\delta Z(t)]^{3/2}$$

where $G = 3.86R^{ - 0.115} \times 10^{ - 8} {\kern 1pt} {\kern 1pt} {\kern 1pt} {\kern 1pt} (m/N^{2/3} )$ for the worn wheel tread^32^, *R* is the radius of the nominal rolling circle.

According to Kalker’s linear creep theory^33^, *F*_*x*_, *F*_*y*_ and *M*_*z*_ can be determined as follows:

11 $${\kern 1pt} {\kern 1pt} {\kern 1pt} \left\{ \begin{gathered} {\kern 1pt} F_{x} = {\kern 1pt} - f_{11} \xi_{x} \hfill \\ F_{y} = - f_{22} \xi_{y} - f_{23} \xi_{\phi } \hfill \\ M_{z} = f_{23} \xi_{y} - f_{33} \xi_{\phi } \hfill \\ \end{gathered} \right.{\kern 1pt} {\kern 1pt} {\kern 1pt} {\kern 1pt} {\kern 1pt}$$

where $f_{ij}$ is the creep coefficient calculated as follows:

12 $${\kern 1pt} {\kern 1pt} {\kern 1pt} \left\{ \begin{gathered} {\kern 1pt} f_{11} = G_{wr} (ab)C_{11} \hfill \\ f_{22} = G(ab)C_{22} \hfill \\ f_{23} = G(ab)^{3/2} C_{23} \hfill \\ f_{33} = G(ab)^{2} C_{33} \hfill \\ \end{gathered} \right.{\kern 1pt} {\kern 1pt} {\kern 1pt} {\kern 1pt} {\kern 1pt}$$

where *G*_*wr*_ is the shear modulusm; The contact ellipse's main and minor semi-axes are identified as *a* and *b*; *C*_*ij*_ is the dimensionless Kalker coefficient^33^.

### Flexible wheelset model

**Figure 5:**
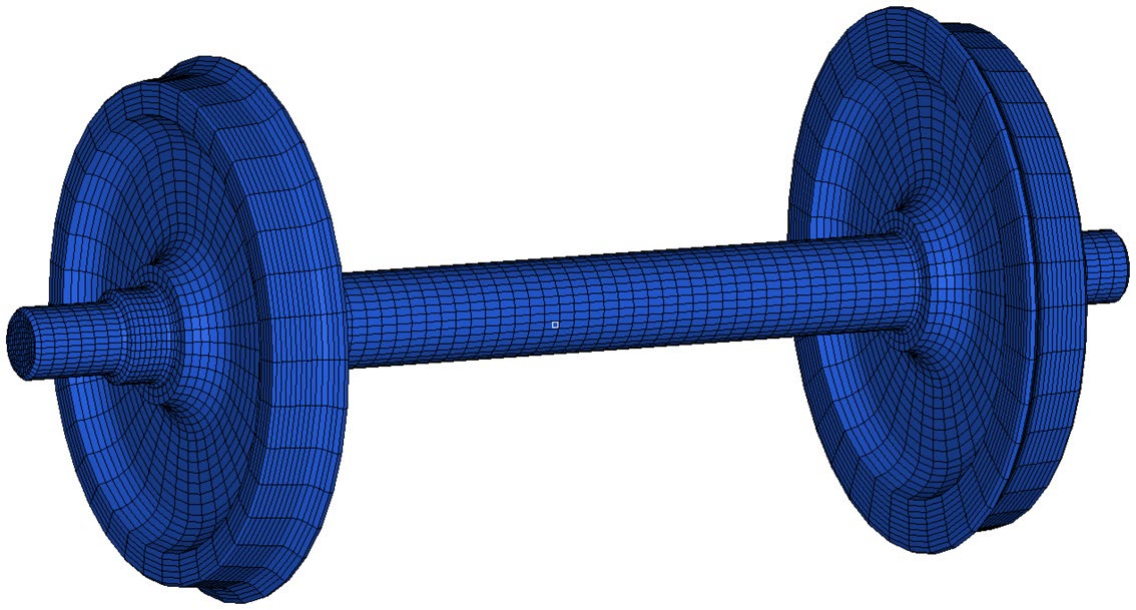
Finite element model of wheelset.

In this study, the flexible deformation of the wheelset is considered in the vehicle model. The flexible wheelset is defined by the finite element (FE) model calculated by ANSYS and is used to determine its modal properties via eigenanalysis. The flexible wheelset model is shown in Fig. 5. The wheelset model is discretized using Solid185 three-dimensional solid elements, with a material Poisson's ratio of 0.3, an elastic modulus of 210 GPa, and a density of 7800 kg/m^3^. The model consists of 104,464 elements and 124,421 nodes. The wheel and axle are treated as a consolidated whole, ignoring the interference-fit relationship between the wheel and axle.The modal vectors are subsequently integrated with the vehicle model by using the finite element multibody system (FEMBS) interface in SIMPACK^34^.

The finite element calculation results of the wheelset, especially the 576.5 Hz modal results, are basically consistent with the relevant research results.^35–38^

### Wheel polygonal model

**Figure 6:**
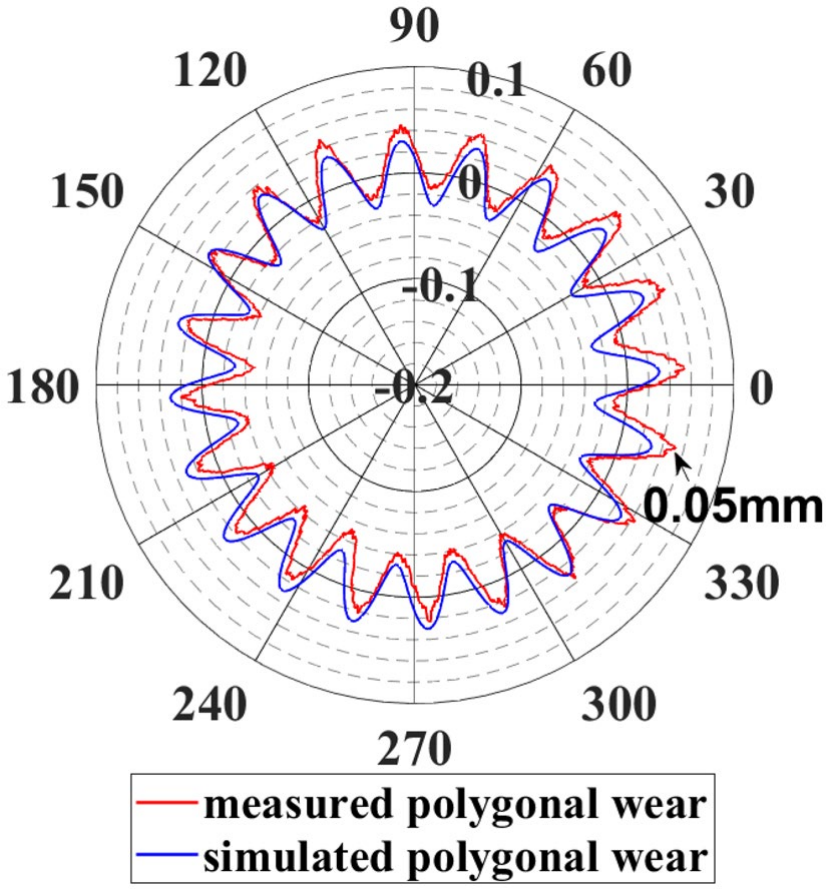
Measured and simulated polygon wear profile of the wheel.

The wheel polygon is caused by uneven tread wear along the circumference while the wheel is being used. The wheel polygon will cause the wheelset to vibrate at high frequencies and induce elastic deformation of the wheelset^37,39–42^. The wheelset’s high-frequency vibration will undoubtedly deteriorate the bearing operating environment since the double-row TRBs are fixed on the end of axle. Figure 6 presents the actual measurement results of a specific type of high-speed train wheel in polar coordinates. The wheel profile exhibits 20th-order polygonal wear characteristics, in which the average wear amplitude is approximately 0.03 mm, and the maximum wear amplitude exceeds 0.05 mm.

To simulate the various amplitudes of wheel polygon wear, harmonic functions can be used to simulate the deviation of wheel diameter:

13 $$\left\{ \begin{gathered} \Delta r = A\sin (N\theta + \theta_{0} ) \hfill \\ r(\theta ) = R - \Delta r \hfill \\ \theta \in {\kern 1pt} {\kern 1pt} {\kern 1pt} {\kern 1pt} [0{\kern 1pt} {\kern 1pt} ,{\kern 1pt} {\kern 1pt} {\kern 1pt} 2\pi ] \hfill \\ \end{gathered} \right.$$

where Δ*r* is the deviation of the wheel diameter along the wheel circumference, *A* is the polygonal wear amplitude, *N* is the polygon order of the wheel, *θ* and *θ*_*0*_ represent the wheel corner and the initial phase angle, respectively. *R* is the nominal rolling radius of the wheel. Modifying the values of wear amplitude *A* and polygon order *N* allows different polygonal wear to be simulated. For instance, Let *A* = 0.03 *mm*, and *N* = 20 to simulate the wheel’s 20th-order polygonal with a wear amplitude of 0.03 mm, as shown in Fig. 6. The simulated polygonal wear, in this case, closely matches the measured wheel profile. The impact of various wear amplitudes *A* on the dynamic responses of TRBs under 20th-order polygons will be investigated in the following.

## Experiment

**Figure 7:**
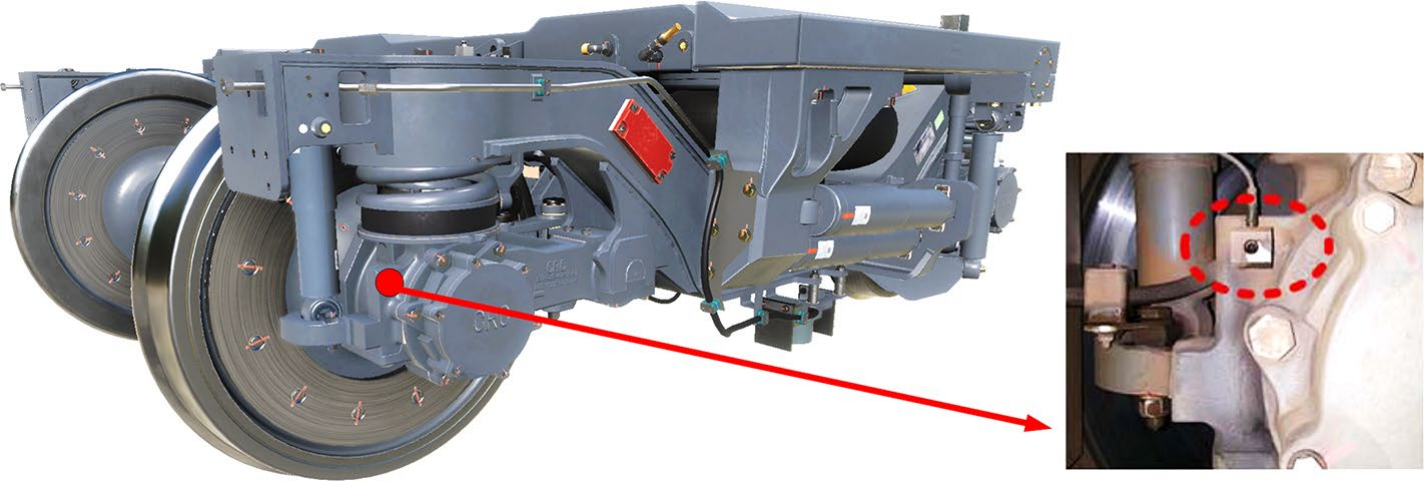
Installation position of acceleration sensor.

We conducted a field test on the railway line connecting Wuhan to Guangzhou to validate the vehicle dynamic model. The structural parameters of the vehicle and bearings for simulation calculations and field test are consistent as shown in Tables 2 and 3 in section "Vehicle dynamic model". The track spectrum used in the simulation is the measured Wuhan-Guangzhou track spectrum, and the field test was also completed on this line. Moreover, the bearings used in the field test are non-faulty bearings and during normal service. Acceleration sensors were installed on the axle box, see Fig. 7, to record the axle box’s vibration acceleration. Then we compared the collected acceleration data with the simulation results when *v* = 300 km/h.

**Figure 8:**
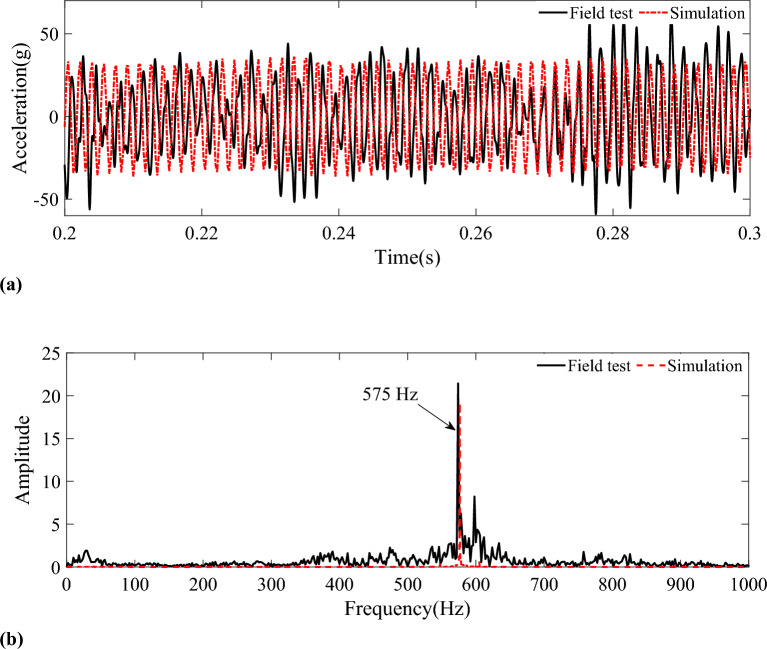
Vertical acceleration of axle box. (a) Vertical acceleration measured by field test and simulation. (b) Frequency spectrum.

Figure 8a shows the vertical acceleration measured by field test and simulation, there is a difference between simulated acceleration and measured acceleration. This is because the simulation vehicle and the field test vehicle are probably located at different positions on the line at the time, the track excitation received by the vehicle at the moment is also different. However, the acceleration amplitude of vertical vibration measured by the simulation and field test is relatively close and within the allowable error range. The RMS acceleration calculated by simulation is 22.9 g, and the measured by field test is 24.3 g. The differences may be due to the simulation model ignoring practical factors, such as flexible deformation of wheelsets, wind, rail corrugation, and simplification of vehicle model. Figure 8b shows the most important frequency characteristic caused by the 20th-order polygonal wear of the wheel: the periodic 575 Hz impact. The main frequency obtained by simulation and experiment is similar. Tiny discrepancy in the frequency of acceleration between the simulation and field test is inevitable due to the omission of practical factors.

In conclusion, the vehicle dynamics model can accurately depict the axle box's actual vibration, validating the model.

## Simulation results

### Effect of wheel polygon on dynamic responses of the axle box bearing

**Figure 9:**
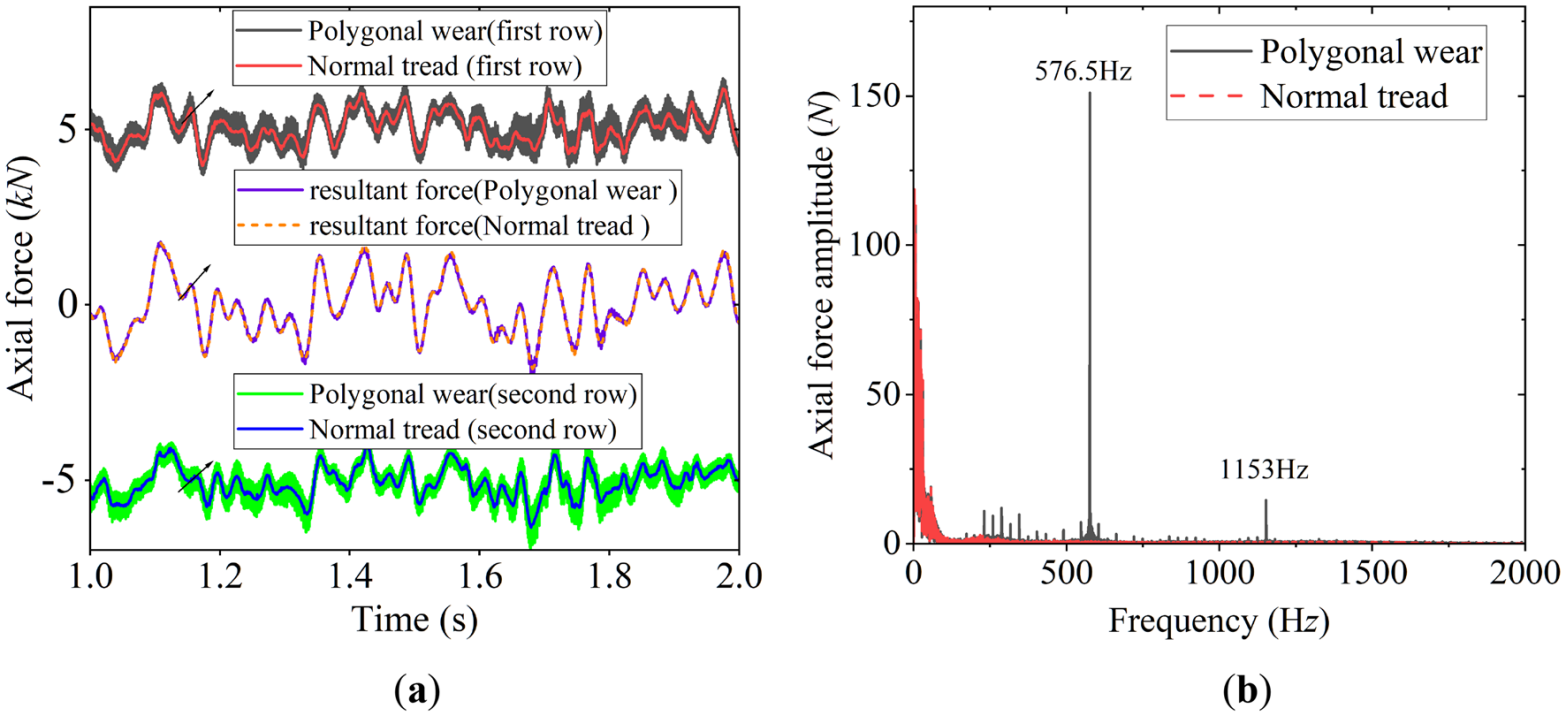
Axial force of double-row TRB: (**a**) time history of axial force; (**b**) frequency spectrum of axial force.

**Figure 10:**
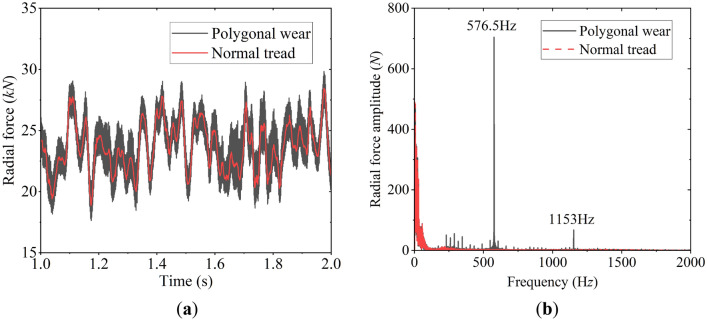
Radial force of double-row TRB: (**a**) time history of radial force; (**b**) frequency spectrum of radial force.

**Figure 11:**
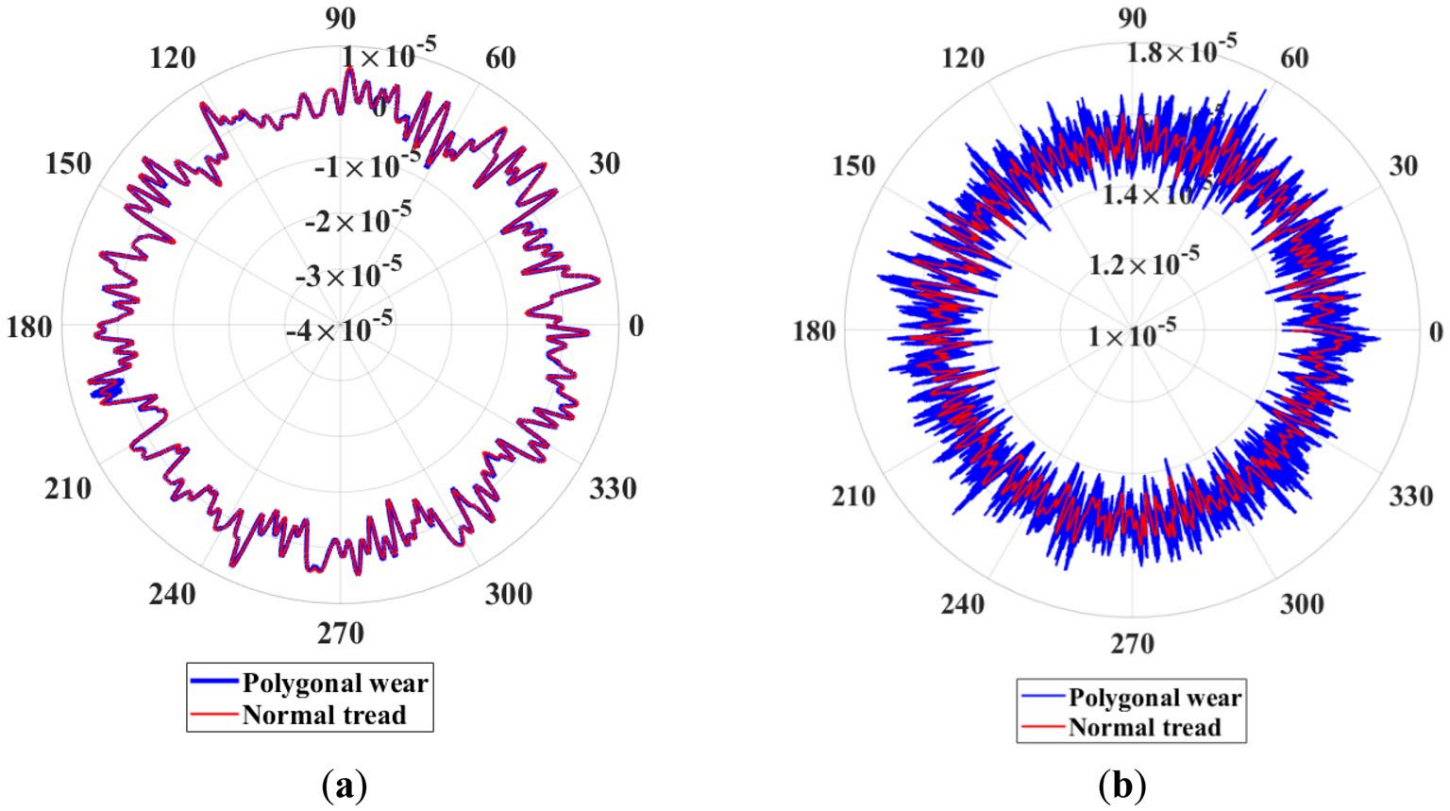
Vibration of bearing centroid: (**a**) axial vibration; (**b**) radial vibration.

To make the simulation more realistic, the measured wheel polygon wear profile (Fig. 6) was used to define the wheel in the model. According to the simulation condition, the vehicle runs on the straight track at *v* = 300 km/h. The track irregularities set in the model are defined by the measured Wuhan–Guangzhou track spectrum, and the model adopts rigid wheelsets. The results of the simulation were compared with those of the normal wheel model. See Figs. 9, 10 and 11.

Figure 9a shows the axial force of double-row TRB. The axial force of the two-row bearing is opposite in direction and has obvious high-frequency vibration components caused by wheel polygonal excitation. Figure 9b shows the spectrum analysis result. The main frequency components include three parts: (1) the low-frequency part, less than 100 Hz, caused by the track irregularity; (2) the wheel 20th-order polygon wear’s excitation frequency of 576.5 Hz when *v* = 300 km/h; (3) the double frequency 1153 Hz of the 576.5 Hz.

Figure 10 shows the radial force of double-row TRB. Compared with the normal wheel, the polygonal excitation of the wheel causes obvious high-frequency vibration and increases the vibration amplitude. Similar to the spectrum analysis results shown in Fig. 9b, the polygon excitation frequency of 576.5 Hz and 1153 Hz is the main frequency of the radial force spectrum.

The wheel polygon excitation increases the vibration of the bearing centroid, see Fig. 11. Therefore, the wheel polygonal wear influences the bearing’s centroid vibration and the rollers’ contact force, which cannot be ignored. Additionally, the high-frequency vibration causes the wheelset’s elastic deformation and affects the bearing’s internal contact force, which will discuss in the next section.

### Effect of flexible deformation of the wheelset on dynamic responses of axle box bearing

To investigate the effects of the wheelset's elastic deformation on the double-row TRB's dynamic responses, we replaced the rigid wheelset with a flexible one in the model. According to the simulation condition, the vehicle runs straightly at *v* = 300 km/h. The track irregularities set in the model are defined by the measured Wuhan–Guangzhou track spectrum. The model adopts normal wheel tread. Then we compared the flexible and rigid wheelset simulation results.

**Figure 12:**
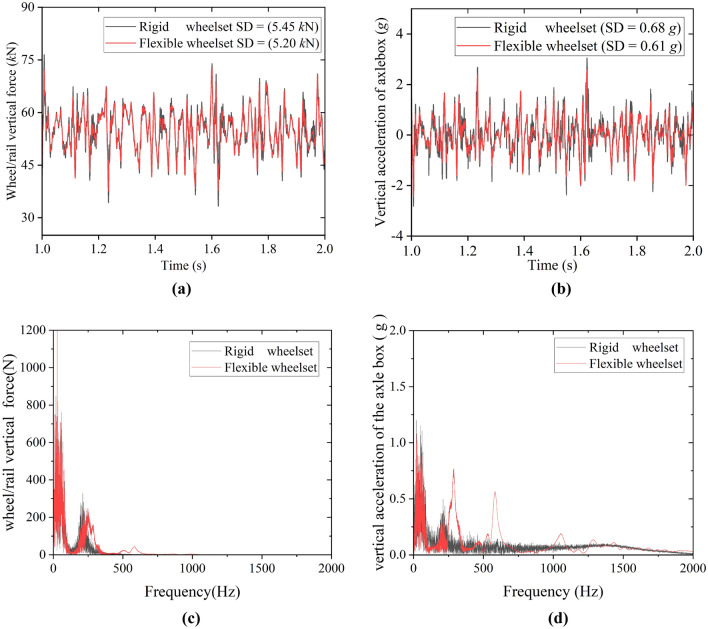
The environmental vibration of the double-row TRBs: (**a**) Wheel/rail vertical force; (**b**) Vertical acceleration of axle box (**c**) Frequency spectrum of wheel/rail vertical force; (**d**) Frequency spectrum of vertical acceleration of axle box.

Figure 12a,b show the simulation results of vertical wheel/rail force and the axle box’s vertical acceleration, respectively. They can describe the double-row TRBs' ambient vibration. The flexible wheelset's vertical wheel/rail force and vertical acceleration of the axle box are smaller than those of the rigid wheelset. Their standard deviation (SD) values in this simulation condition decreased slightly, respectively. Figure 12c,d show the spectrum analysis results of vertical wheel/rail force and the axle box’s vertical acceleration, respectively. The results of the flexible wheelset model and the rigid wheelset model are consistent, with the main frequency being the frequency of track irregularity excitation. However, the spectrum of the flexible wheelset model has richer high-frequency characteristics than the spectrum of the rigid wheelset model.

**Figure 13:**
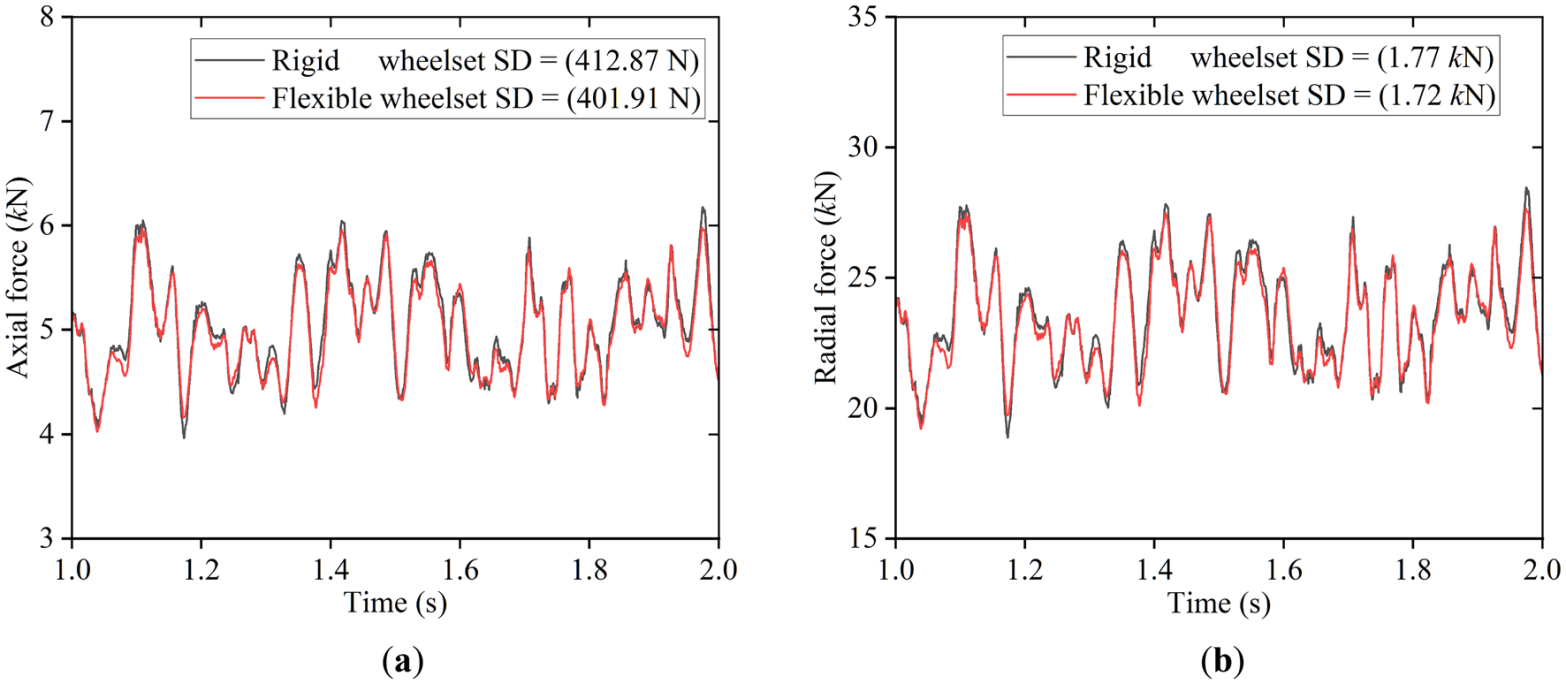
Contact force of the bearing rollers: (**a**) axial force; (**b**) radial force.

Figure 13 shows the results of the axial and radial force of the double-row TRB. The SD value of both the axial and radial force of the flexible wheelset in this simulation condition decreased compared with that of the rigid wheelset. The abovementioned results show that the flexible deformation of the wheelset plays a buffering role in the vehicle operation, alleviating the wheel/rail impact and reducing the axle box’s vertical vibration and the rollers’ contact force of the double-row TRB.

To determine how the double-row TRBs' dynamic responses are affected by the flexible wheelset with polygonal tread, we replaced the normal wheel with the measured polygon wear profile (Fig. 6) in the model.

**Figure 14:**
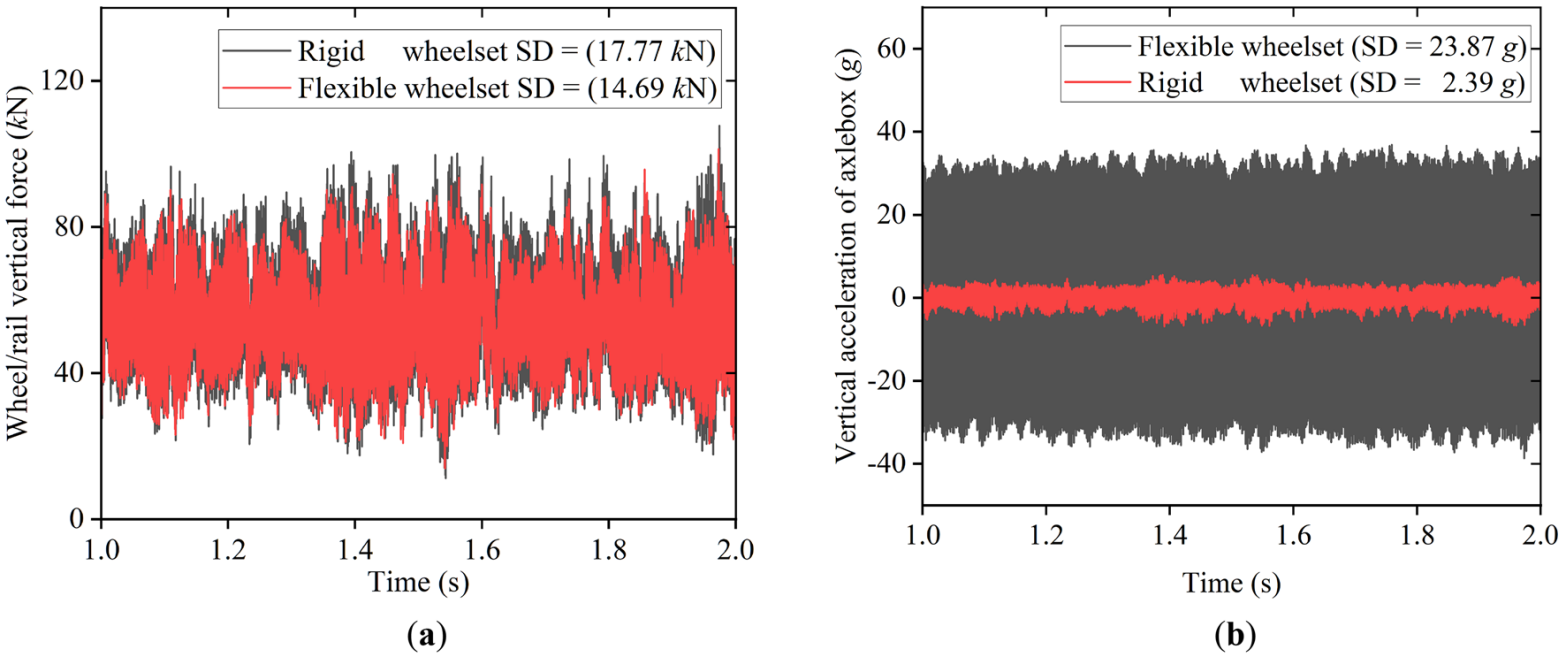
The environmental vibration of the double-row TRBs: (**a**) Wheel/rail vertical force; (**b**) Vertical acceleration of axle box.

Figure 14a shows the wheel/rail vertical force. In this simulation condition, the SD value of the flexible wheelset decreased compared with that of the rigid wheelset, indicating that the wheel/rail impact caused by wheel polygonal wear can be reduced by the wheelset's flexibility.

However, the flexible wheelset with polygonal tread cannot alleviate the axle box’s vertical vibration but instead increases the SD value of the axle box’s vertical acceleration by about 9 times, see Fig. 14b. This is because when the vehicle is running at *v* = 300 km/h, the excitation frequency caused by the wheel's 20th-order polygon is 576.5 Hz, and the flexible wheelset's 20th-order modal frequency is 577 Hz. The two frequencies are similar. At this time, the wheelset resonates, and the elastic deformation of the shaft ends increases significantly. Since the double-row TRB secures the axle box to the shaft ends, the axle box’s vertical acceleration increases significantly.

**Figure 15:**
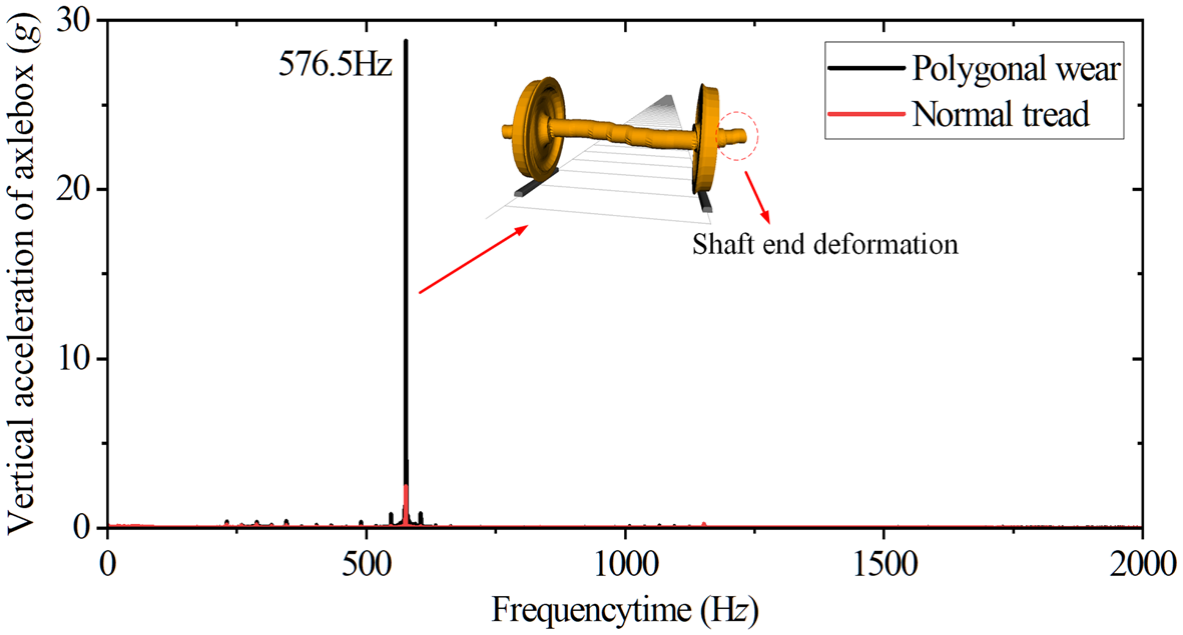
Frequency spectrum.

The frequency spectrum analysis results of the axle box’s vertical acceleration are shown in Fig. 15. The main frequency is still 576.5 Hz, which verifies the above conclusion.

**Figure 16:**
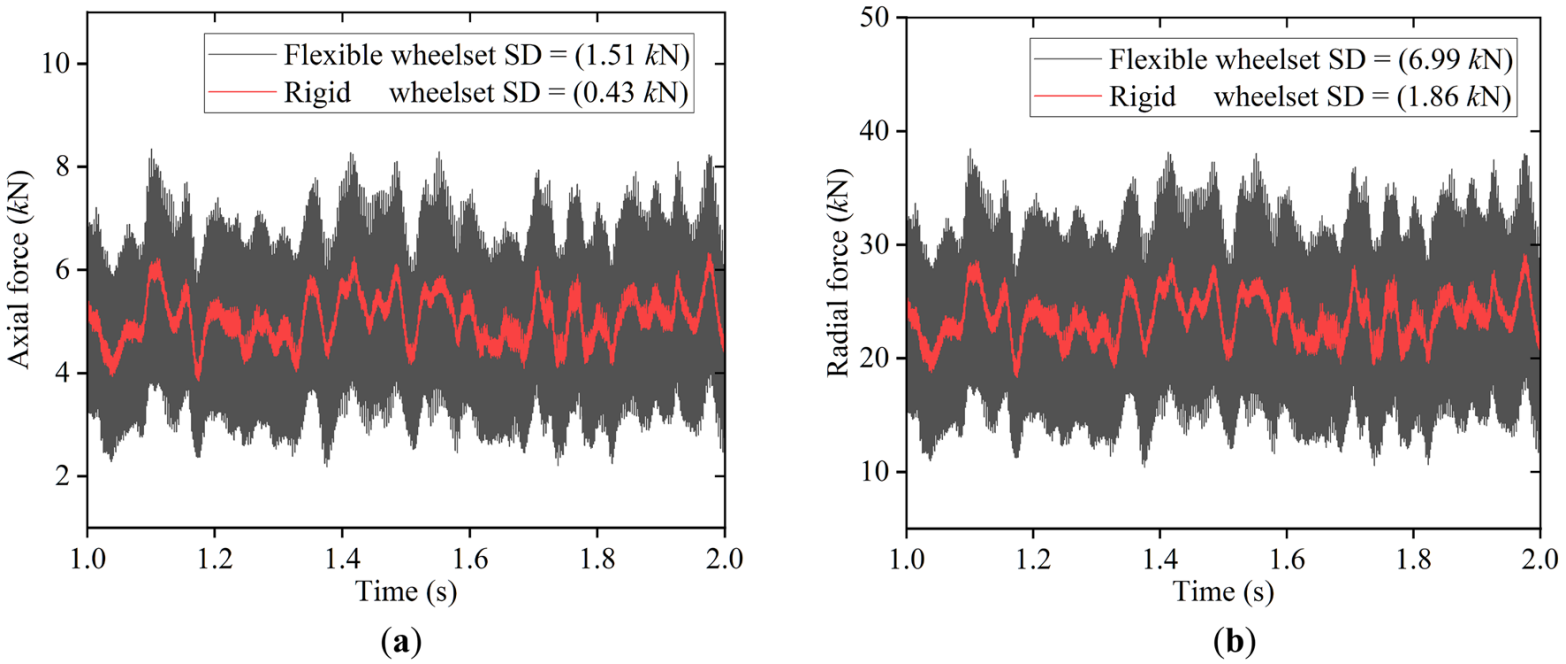
Contact force of the bearing rollers: (**a**) axial force; (**b**) radial force.

Figure 16 shows the simulation results for the double-row TRB’s axial and radial forces. In this simulation condition, the SD value of the axial force of the flexible wheelset increased significantly compared with that of the rigid wheelset. The SD value of the radial force also increased significantly. The above calculation results show that the elastic deformation of the shaft end will increase the internal contact forces of the double-row TRB.

In summary, for the normal wheel, the flexible wheelset’s elastic deformation can alleviate the wheel/rail impact, reduce the axle box’s vertical vibration, and decries the axle box bearing’s internal contact force. However, when the wheel has 20th-order polygon wear, the high-frequency vibration excited by the polygonal wear will increase the wheelset’s local elastic deformation, such as the shaft end, which will deteriorate the operation environment of the bearings. Lead to an increase in bearings’ internal contact forces, and reduce the reliability of the bearing.

### Effect of different vehicle velocity on the dynamic response of axle box bearings

The excitation frequency of the wheel polygon is related to the vehicle velocity. The velocity was set to vary from 50 to 350 km/h in the rigid and flexible wheelset models to verify the dynamic responses of the double-row TRBs under different vehicle velocities. The other simulation conditions required that the vehicle runs on a straight track, which applies the measured Wuhan–Guangzhou track spectrum, and the measured wheel polygon wear profile (Fig. 6) was used to define the wheel in the model.

**Figure 17:**
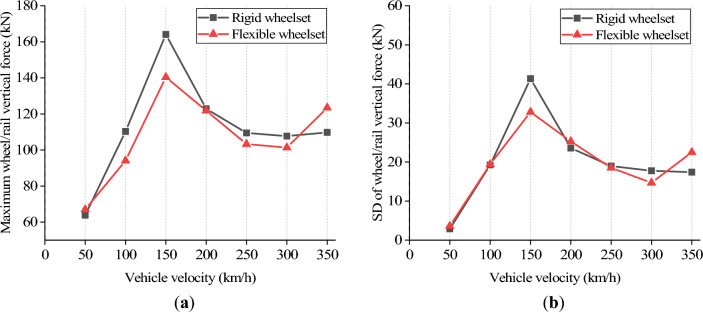
Wheel/rail vertical force at different vehicle velocity: (**a**) maximum value; (**b**) standard deviation value.

Figure 17 shows the wheel/rail vertical force at various velocities. Both the rigid and flexible wheelsets’ wheel/rail vertical forces follow a consistent variation law that peaks at *v* = 150 km/h rather than increasing with velocity, which agrees with the findings in the literature^34^. Additionally, when *v* < 300 km/h, the rigid wheelset's vertical wheel/rail force is greater than that of the flexible wheelset's, since the flexible wheelset can alleviate the wheel/rail impact. However, when *v* reaches 350 km/h, the flexible wheelset’s wheel/rail vertical force increases significantly and exceeds that of the rigid wheelset. This is due to the fact that when velocity increases, the high-frequency vibration of the wheelset increases.

**Figure 18:**
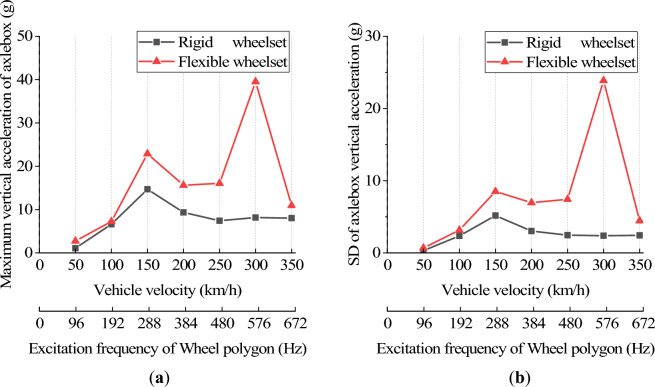
Vertical acceleration of axle box at different vehicle velocity: (**a**) maximum value; (**b**) standard deviation value.

Figure 18 shows axle box’s the vertical acceleration at different vehicle velocities. The flexible wheelset’s axle box vertical acceleration is greater than the rigid wheelset’s within 350 km/h. Two peaks appear at *v* = 150 and 300 km/h. Notably, the axle box’s vertical acceleration increases significantly when *v* = 300 km/h. This is due to the 20th-order polygon wheel’s excitation frequency at 300 km/h being 576.5 Hz. During this time, the wheelset resonates, and the shaft end’s elastic deformation increases, increasing the axle box’s vertical acceleration significantly.

**Figure 19:**
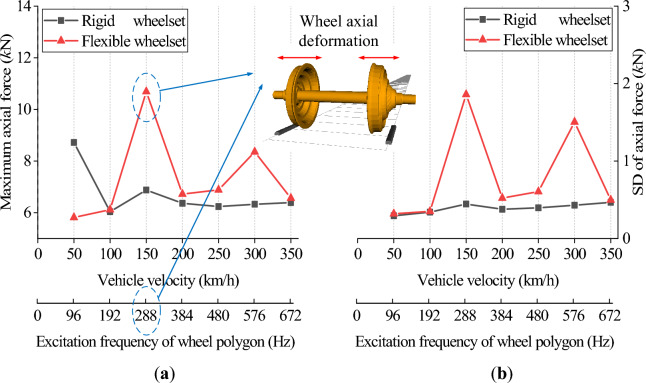
Axial contact force at different vehicle velocity: (**a**) maximum value; (**b**) standard deviation value.

**Figure 20:**
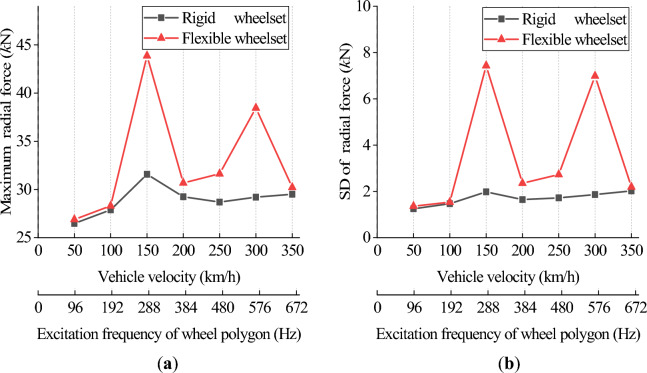
Radial contact force at different vehicle velocity: (**a**) maximum value; (**b**) standard deviation value.

Figures 19 and 20 show the simulation results of the double-row TRB’s axial and radial contact force. The flexible wheelset’s axial and radial contact forces of the double-row TRB are greater than that of the rigid wheelset, and the peak values appear when *v* = 150 and 300 km/h.

Different from the results of axle box’s vertical acceleration, the double-row TRB’s axial and radial contact forces at *v* = 150 km/h are significantly greater than those at *v* = 300 km/h. Through analysis, we found that the polygonal wheel’s excitation frequency is 288 Hz when *v* = 150 km/h, near the wheel’s 12th-order modal frequency, i.e., 274 Hz. During this time, the wheelset resonates, and the axial elastic deformation of the wheel increases significantly (Fig. 19), which increases the wheelset’s lateral vibration and the double-row TRB’s axial force. Simultaneously, under the action of tapered rollers, the increase in axial force will directly lead to an increase in radial contact force (Fig. 20).

### Effect of wheel polygon amplitude on the dynamic response of axle box bearings

We set the wheel polygon wear amplitudes *A* ranging from 0.01 to 0.05 mm in the model, using the simulation method mentioned in Section "Wheel polygonal model", to analyze the the double-row TRB’s dynamic responses under different wheel polygon wear amplitudes. The other simulation conditions require that the vehicle runs on a straight track at velocities of 50–300 km/h, and the measured Wuhan–Guangzhou track spectrum is applied. The SD values of wheel/rail vertical force, vertical acceleration of axle box, the double-row TRB’s axial and radial force, and maximum contact stress of the double-row TRB at different vehicle velocities and wheel polygon wear amplitudes were comprehensively analyzed.

**Figure 21:**
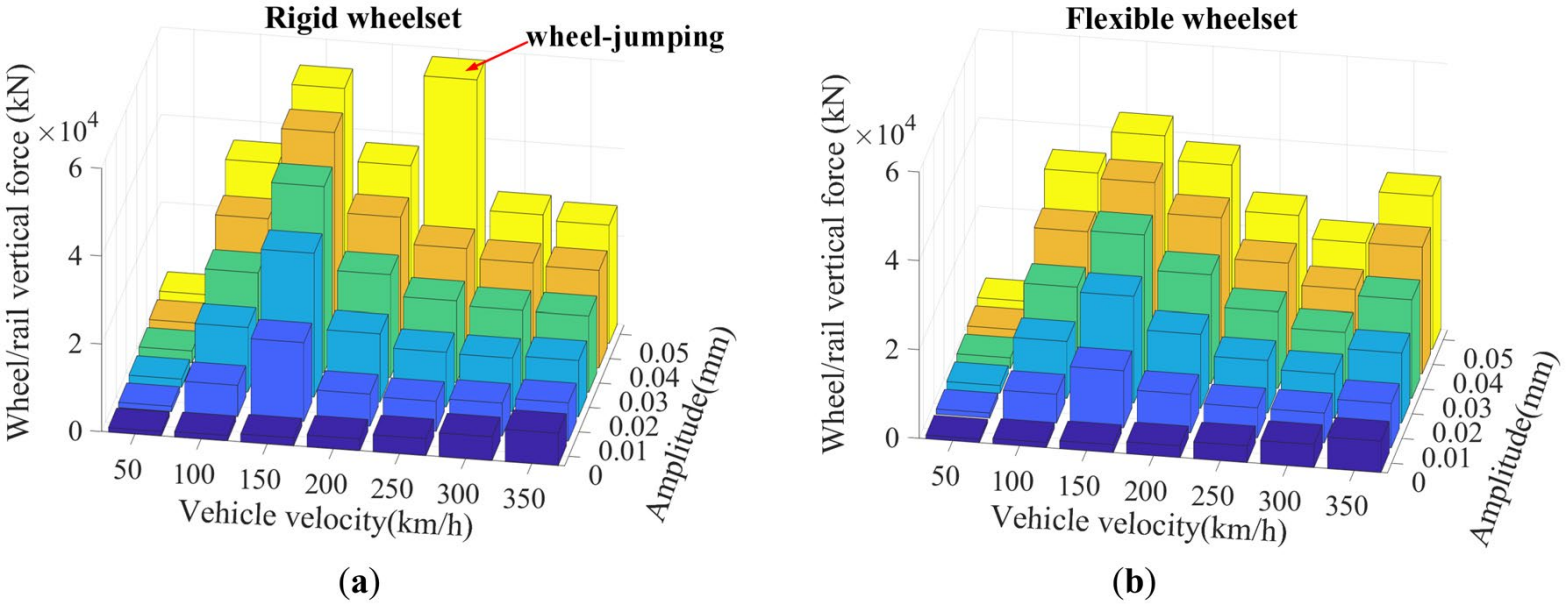
SD values of wheel/rail vertical force: (**a**) rigid wheelset model; (**b**) flexible wheelset model value.

Figure 21 shows the SD values of the wheel/rail vertical force, which indicates that the wheel/rail vertical force of the rigid and the flexible wheelset model change with vehicle velocities and the wheel polygon amplitudes are generally the same. At the same vehicle velocity, the wheel/rail vertical force increases with the increase in wheel polygon wear amplitude. Moreover, the changing rule of wheel/rail vertical force at different vehicle velocities for the same polygonal wear amplitude of the wheel are consistent with the results given in Fig. 17 in Section "Effect of different vehicle velocity on the dynamic response of axle box bearings". It is worth noting that when *v* = 250 km/h and *A* = 0.05 mm, the rigid wheelset’s wheel/rail vertical force increases significantly, which is caused by the wheel jumping.

**Figure 22:**
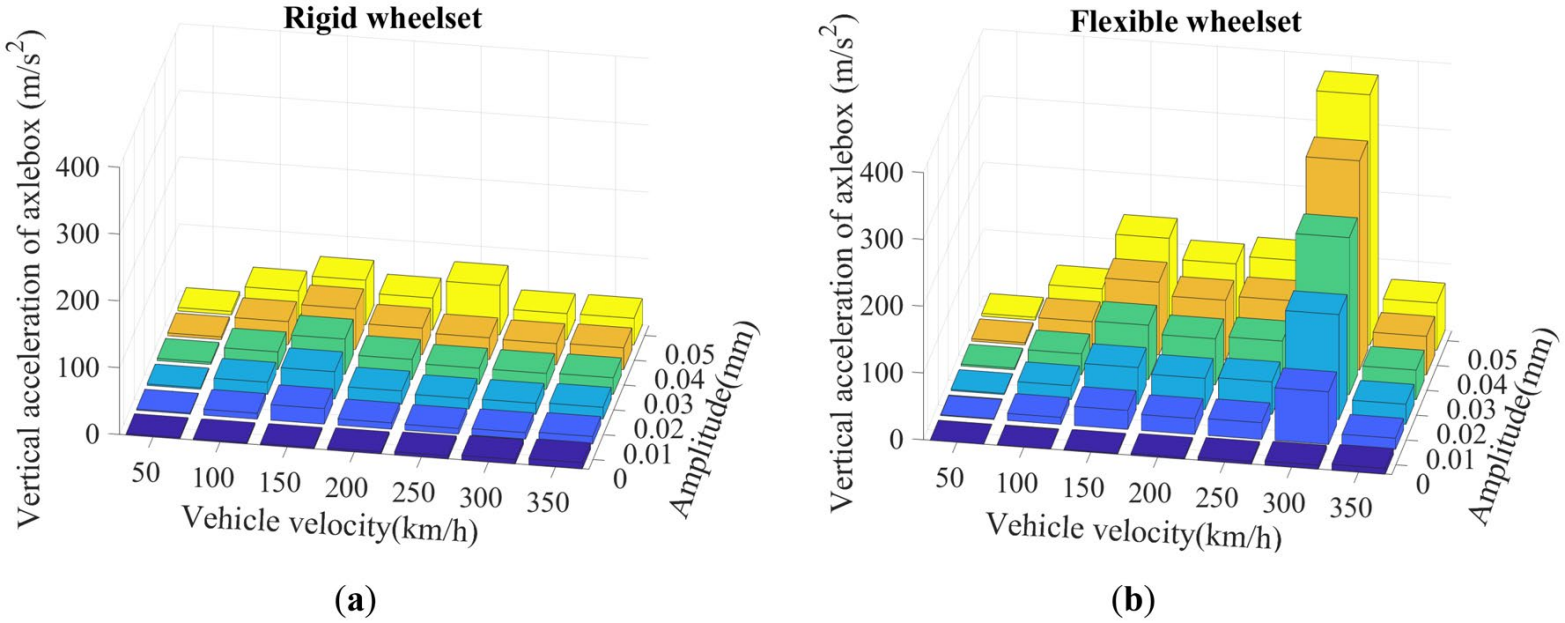
SD values of vertical acceleration of axle box: (**a**) rigid wheelset model; (**b**) flexible wheelset model value.

Figure 22 shows the SD values of the axle box’s vertical acceleration. The flexible wheelset model’s vertical acceleration amplitude is significantly greater than rigid wheelset, and the variation law is the same. At the same vehicle velocity, the axle box’s vertical acceleration increases with the increase in wheel polygon wear amplitude. At the same wheel polygonal wear amplitude, two peaks of vertical acceleration appear at *v* = 150 and 300 km/h. This is due to the wheelset resonance caused by the excitation frequency of 576.5 Hz of the wheel’s 20th-order polygon at *v* = 300 km/h, consistent with the results shown in Fig. 15 in Section "Effect of flexible deformation of the wheelset on dynamic responses of axle box bearing".

**Figure 23:**
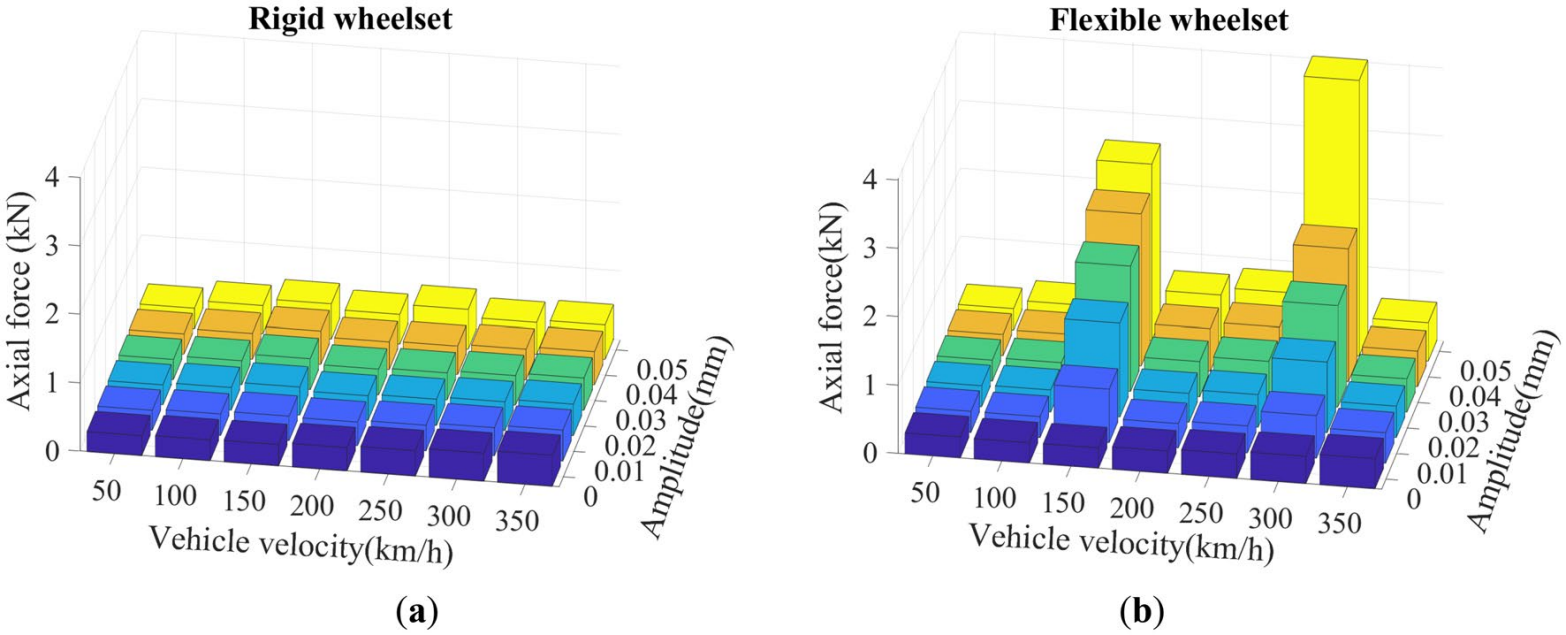
SD values of axial contact force of the double-row TRB: (**a**) rigid wheelset model; (**b**) flexible wheelset model value.

**Figure 24:**
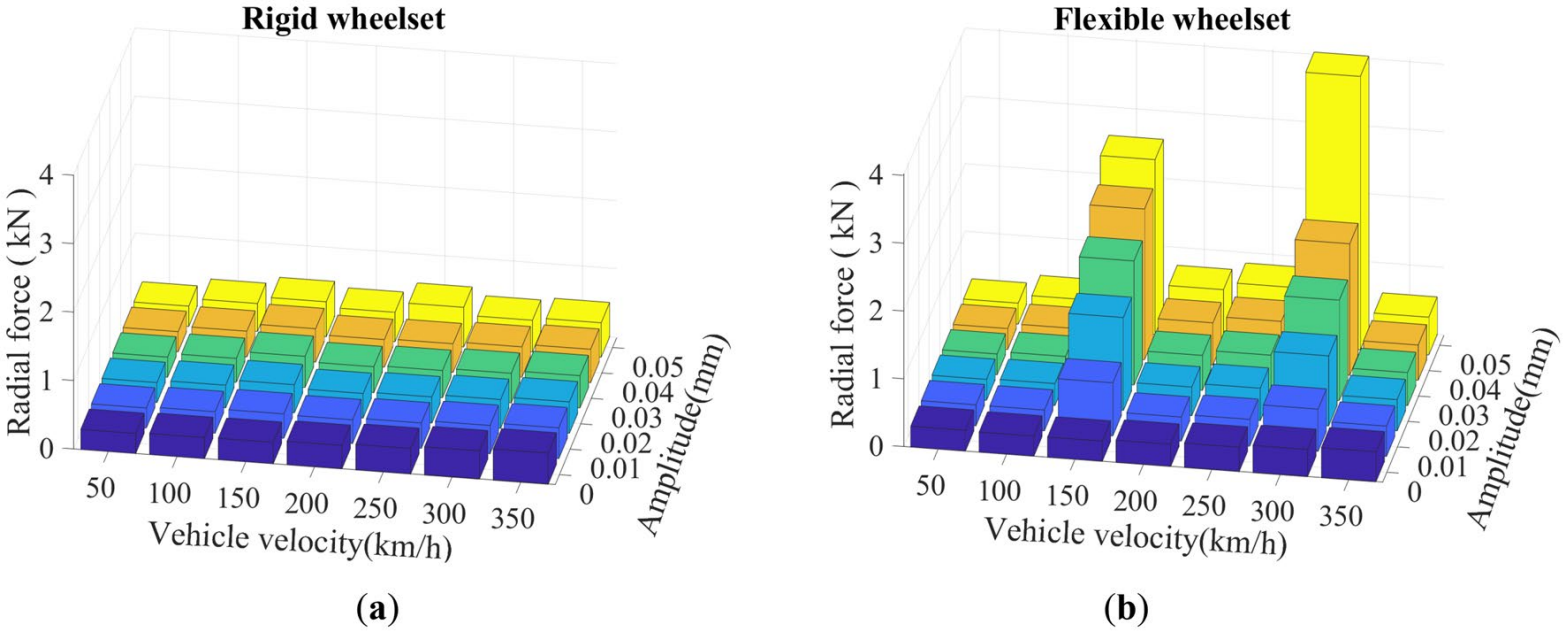
SD values of radial contact force of the double-row TRB: (**a**) rigid wheelset model; (**b**) flexible wheelset model value.

The SD values of the double-row TRB’s axial and radial contact forces are shown in Figs. 23 and 24. The variation law of the double-row TRB’s axial and radial force is the same. At the same vehicle velocity, the axial and radial force increase slightly with the increase in wheel polygon wear amplitude. Furthermore, at the same wheel polygonal wear amplitude, the axial and radial force increased significantly at *v* = 150 and 300 km/h. This is due to the resonance of wheelset caused by 20th-order polygonal wheel’s excitation frequency of 288 Hz and 576.5 Hz at the vehicle velocities of 150 km/h and 300 km/h, respectively. The results are consistent with the conclusions mentioned in Sections "Effect of flexible deformation of the wheelset on dynamic responses of axle box bearing" and "Effect of different vehicle velocity on the dynamic response of axle box bearings".

**Figure 25:**
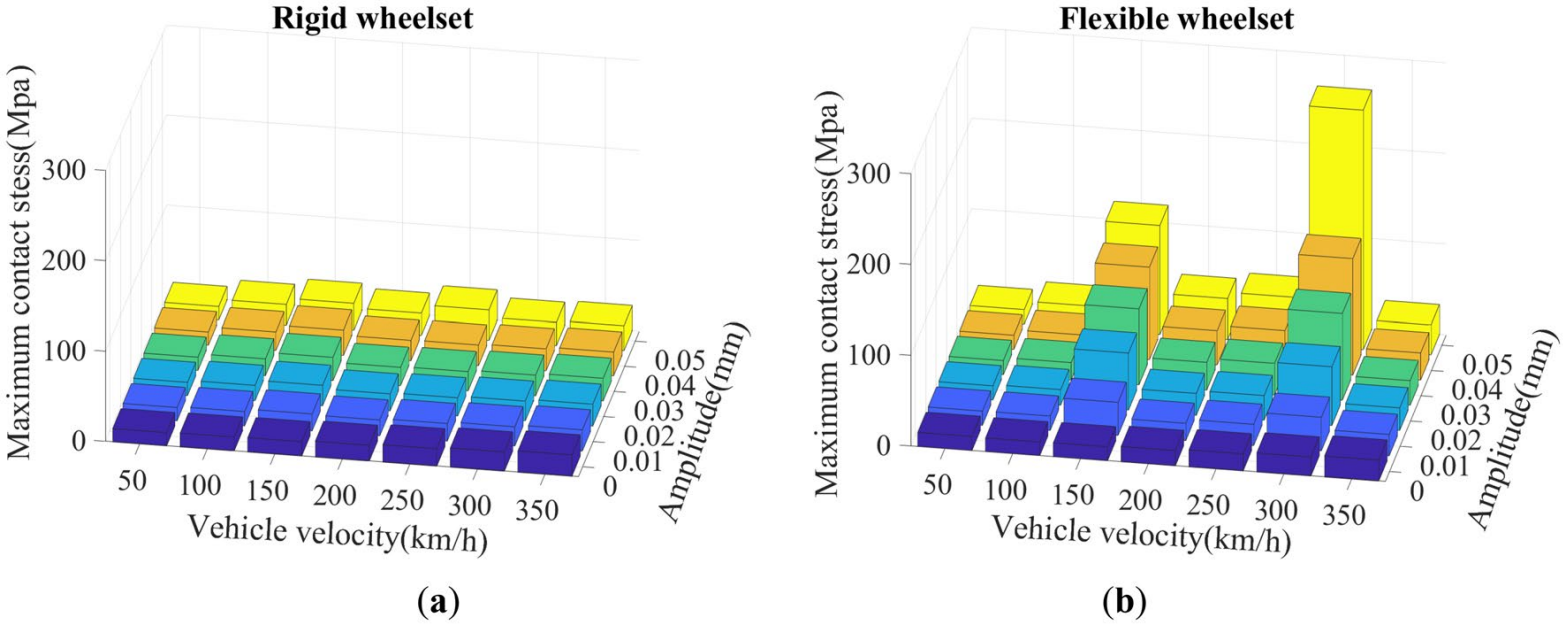
SD values of maximum contact stress of the double-row TRB: (**a**) rigid wheelset model; (**b**) flexible wheelset model value.

Figure 25 shows the double-row TRB’s maximum contact stress. The variation rule of maximum contact stress is consistent with the variation rule of the axial and radial forces, as shown in Figs. 23 and 24.

## Conclusions

On the basis of traditional vehicle dynamics theory, this article analyzes the dynamic response of axle box bearings for high-speed trains, considering wheelset flexibility and polygonal wear. The following conclusions are obtained through simulation calculations, which provide a reference for further analysis of axle box bearing life and fatigue failure.

1. The wheel polygon excitation of the rigid wheelset increases the double-row TRB’s vibration and the rollers’ contact force. This gives rise to high-frequency vibration components, which cannot be ignored.
2. The elastic deformation of the flexible wheelset with normal wheel tread can alleviate the wheel/rail impact. In the simulation condition, when *v* = 300 km/h, the SD values of the wheel/rail vertical contact force, axle box vertical acceleration, and the double-row TRB’s axial and radial forces decreased slightly, respectively.
3. When the flexible wheelset and the measured wheel’s 20th-order polygon profile are considered simultaneously at *v* = 300 km/h in the simulation condition, the SD value of the flexible wheelset’s wheel/rail vertical contact force decreased compared with that of the rigid wheelset. Contrariwise, the SD values of the axle box’s vertical acceleration increased by about 9 times, and the double-row TRB’s axial and radial contact forces increased significantly, respectively.
4. The wheel/rail vertical force reached the peak value when *v* = 150 km/h, and the flexible wheelset’s peak value was smaller. The vertical acceleration of the axle box for the flexible wheelset was greater than the rigid wheelset. When *v* = 300 km/h, the axle box’s vertical vibration increases significantly. The double-row TRB’s contact forces of the flexible wheelset are greater and the axial and radial forces reach their peak when *v* = 150 km/h.
5. When the vehicle velocity varies from 50 to 350 km/h and the polygon wear amplitude of wheel varies from 0.01 to 0.05 mm, the double-row TRB’s dynamic response is summarized as follows. When the vehicle velocity is constant, the SD value of the wheel/rail vertical force, vertical acceleration of the axle box, bearing contact force, and maximum contact stress increase with the increase in the polygonal wear amplitude *A*. When the polygonal wear amplitude is constant, the variation laws of the abovementioned SD values are consistent with conclusion (4).

## Data Availability

All data generated or analysed during this study are included in this published article.
